# Molecular Monolayer Sensing Using Surface Plasmon Resonance and Angular Goos-Hänchen Shift

**DOI:** 10.3390/s21134593

**Published:** 2021-07-05

**Authors:** Cherrie May Olaya, Norihiko Hayazawa, Maria Vanessa Balois-Oguchi, Nathaniel Hermosa, Takuo Tanaka

**Affiliations:** 1 National Institute of Physics, University of the Philippines Diliman, Quezon City 1101, Philippines; cherriemay.olaya@riken.jp (C.M.O.); hayazawa@riken.jp (N.H.); nhermosa@nip.upd.edu.ph (N.H.); 2 Innovative Photon Manipulation Research Team, RIKEN Center for Advanced Photonics, Wako 351-0198, Japan; mariavanessa.balois@riken.jp; 3 Metamaterials Laboratory, RIKEN Cluster for Pioneering Research, Wako 351-0198, Japan

**Keywords:** surface plasmon resonance, goos-hänchen shift, fresnel, plasmon, self-assembled monolayer

## Abstract

We demonstrate potential molecular monolayer detection using measurements of surface plasmon resonance (SPR) and angular Goos-Hänchen (GH) shift. Here, the molecular monolayer of interest is a benzenethiol self-assembled monolayer (BT-SAM) adsorbed on a gold (Au) substrate. Excitation of surface plasmons enhanced the GH shift which was dominated by angular GH shift because we focused the incident beam to a small beam waist making spatial GH shift negligible. For measurements in ambient, the presence of BT-SAM on a Au substrate induces hydrophobicity which decreases the likelihood of contamination on the surface allowing for molecular monolayer sensing. This is in contrast to the hydrophilic nature of a clean Au surface that is highly susceptible to contamination. Since our measurements were made in ambient, larger SPR angle than the expected value was measured due to the contamination in the Au substrate. In contrast, the SPR angle was smaller when BT-SAM coated the Au substrate due to the minimization of contaminants brought about by Au surface modification. Detection of the molecular monolayer acounts for the small change in the SPR angle from the expected value.

## 1. Introduction

Goos-Hänchen (GH) shift is a diffractive correction to the reflection coefficient that results from the interaction of an optical beam and a planar interface [1]. The components of the wave vector undergo different phase and amplitude changes after reflection. When these wave vector components recombine, a lateral shift and a tilt are induced in the reflected beam with respect to the values predicted by geometric optics [2,3,4]. The lateral shift, which is also called spatial GH ($\Delta_{GH}$) shift, results from the phase changes in the wave vector components. Based on the stationary phase method, Artmann’s formulation showed that $\Delta_{GH}$ is proportional to the angular derivative of the phase of the complex reflectivity [2]. The tilt in the reflected beam, termed angular GH ($\Theta_{GH}$) shift, results from the amplitude changes in the wave vector components. The change in the amplitude leads to a distortion of the reflected beam inducing a propagation-dependent deflection [5,6,7]. $\Theta_{GH}$ is proportional to the angular derivative of the amplitude of complex reflectivity [8]. The reflected beam, then, experiences a total GH shift that is the linear combination of $\Delta_{GH}$ and $\Theta_{GH}$ [9]. Typically, GH shift is of the same order of magnitude as the incident source. As such, enhancement is necessary for any practical sensing purposes. Excitation of surface plasmons is one way of enhancing the GH shift, and GH shift measurement at surface plasmon resonance (SPR) has been shown to have great potential in high sensitivity refractive index (RI) sensing [10,11,12,13].

The majority of the studies involving SPR-enhanced GH shift measurements primarily focuses on the measurement of $\Delta_{GH}$ enhanced by the abrupt phase jump at the SPR angle [10,11,12,13]. We deem, however, that SPR-enhanced $\Theta_{GH}$ could provide larger enhancement of the GH shift. The distinct property of SPR aside from the abrupt phase jump is the sharp reflectivity dip at the SPR angle, which is ideal for $\Theta_{GH}$. Furthermore, Aiello et al., in Ref. [14], discussed how beam propagation affects the magnitude of $\Theta_{GH}$ measured at the detector. Essentially, in our case, three factors contribute to the amount of $\Theta_{GH}$ measured: (1) excitation of surface plasmons which determines the amount of reflectivity dip; (2) size of the minimum beam waist; and (3) propagation distance of the reflected beam. While measurement of SPR-enhanced $\Delta_{GH}$ is determined only by the first factor, the other two allow for even further enhancement of the GH shift measured through $\Theta_{GH}$ [14]. This would warrant more control on the amount of enhancement necessary for a particular measurement.

Previously, in Ref. [15], we demonstrated SPR-enhanced $\Theta_{GH}$-dominated GH shift measurement as a potential RI sensor using gold (Au) substrate. However, the surface property of Au has been met with controversy as to whether Au surface is hydrophobic or hydrophilic. Although it has been shown that bare Au is hydrophilic, the Au surface can be immediately covered by carbon and oxygen contaminants—a monolayer of which results in a hydrophobic Au surface [16]. In this work, to regulate Au surface properties in ambient, we employed a hydrophobic molecular monolayer to inhibit surface contamination on the substrate. We used a self-assembled monolayer (SAM) of benzenethiol (BT) formed on the Au surface and titanium (Ti) as the adhesion layer on a glass substrate. For a more accurate modeling of the experimental system, we took into account both the Au and Ti layers, as well as the focused incident beam, in the analysis. The choice of BT as a test molecule comes from the S-Au bond formed between the Au substrate and the BT molecule ensuring adsorption of the molecule to the substrate. Moreover, since BT is a small molecule, BT-SAM formation actually poses a challenge during typical detection schemes. As such, the detection of the presence of BT-SAM is a good indicator of the sensitivity of the potential sensor.

## 2. Surface Plasmon Resonance and Goos-Hänchen Shift of Focused Beam

**Figure 1:**
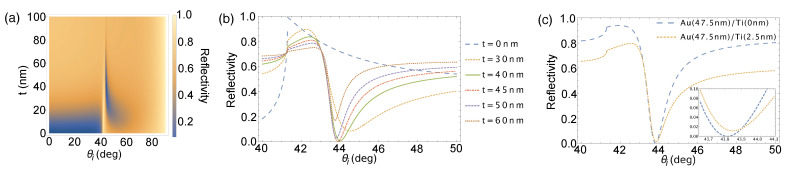
(**a**) Angular reflectivity spectrum for a BK7/Ti/Au/air structure with varying Au film thickness with collimated incident beam for an angular range from normal incidence to grazing incidence; (**b**) representative plots at several Au film thicknesses; (**c**) comparison of reflectivity spectra with (yellow dashed line) and without (blue dashed line) the Ti adhesion layer. The inset shows a magnified area at the reflectivity dip to distinctly see the difference between the two plots.

Measuring the intensity of the reflected beam is a typical way of characterizing the interaction between a monochromatic plane wave and surface plasmons. For multilayered structures, these are calculated using the transfer matrix method (TMM) based on the Fresnel reflection coefficient for thin films [17,18]. A dip in the angular reflectivity spectrum is observed due to the transfer of energy from the incident beam to the surface plasmons, followed by its dissipation into the metal film. The depth of the reflectivity dip depends on the material, thickness, and roughness of the metal film, and it is characterized by its quality factor (Q factor). At the optimum thickness, reflectivity reaches its minimum value indicating strongest surface plasmon excitation [18]. The depth, width, and asymmetry of the reflectivity dip changes as the thickness of the metal film diverges from the optimum metal film thickness. This is shown in the plot in Figure 1a. The structure used for calculation consists of a layer of Au (ε = −11.740 + 1.2611*i* [19]) film with varying thickness $t_{Au}$ on top of a 2.5-nm titanium (Ti, ε = −6.8655 + 20.361*i* [20]) adhesion layer. Both films are on top of a BK7 (n = 1.515) prism. Ti is less absorptive and does not change the structural property of the film making it a good adhesion layer [21,22]. A *p*-polarized laser source (λ = 632.8 nm) impinges on the structure through the BK7 prism at varying angles of incidence, $\theta_{i}$. At $t_{Au}$ = 0, no surface plasmons are excited and the reflectivity spectrum resembles that of a prism-air structure with some deviation caused by the Ti film. As the Au film thickness is increased, the dip in reflectivity becomes more prominent until it reaches the optimum value at $t_{Au}$ = 43.8 nm. Further increase in Au film thickness shows the reflectivity dip becoming shallower and broader until the reflectivity spectra assumes that of a bulk Au mirror. This trend is clearly shown in the angular reflectivity spectra in Figure 1b. The presence of the Ti layer shows almost negligible effect to the location of the SPR angle as shown in Figure 1c.

**Figure 2:**
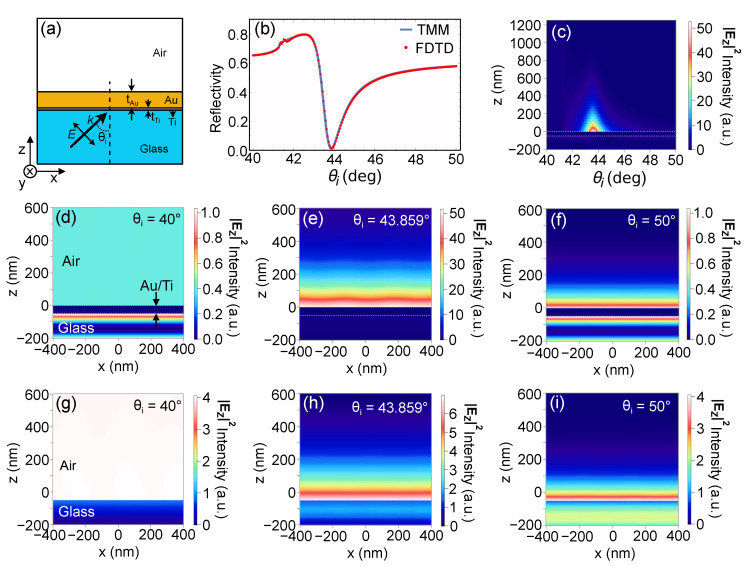
(**a**) Model used for FDTD simulations consisting of Au ($t_{Au}$ = 47.5 nm), Ti ($t_{Ti}$ = 2.5 nm) and glass layers illuminated by a linear *p*-polarized electric field at an incident angle $\theta_{i}$. (**b**) Comparison of reflectivity calculated using FDTD (solid red circles) and analytically using TMM (solid blue line). (**c**) Rapidly decaying electric field at SPR in the z direction at various angles of incidence. Electric field distribution at the (**d**–**f**) BK7/Ti/Au/air and (**g**–**i**) BK7/air interface for: (**d**,**g**) incident angle smaller than the SPR angle ($\theta_{i}$ = 40°); (**e**,**h**) incident angle equal to the SPR angle ($\theta_{i}$ = 43.859°), and (**f**,**i**) incident angle larger than the SPR angle ($\theta_{i}$ = 50°).

In order to visualize the field distribution of the sensing area on the Au substrate, numerical simulations were made using a commercially available finite-difference time-domain (FDTD) software (Lumerical-Ansys, Inc., Vancouver, Canada) to determine the behavior of the reflected light at varying angles of incidence. Figure 2a shows the model used for the simulations. The structure is based on the actual substrate used in the experiments. It consists of a $t_{Au}$ = 47.5 nm thick Au top layer, followed by a $t_{Ti}$ = 2.5 nm thick Ti adhesion layer, and a bulk glass bottom layer whose thickness is much larger than the Au and Ti layers combined. A linearly *p*-polarized electric field (λ = 632.8 nm) was injected into the model at an incident angle $\theta_{i}$ from the glass side. Figure 2b shows the comparison of reflectivity plot generated analytically using TMM and numerically using FDTD. The perfect overlap between the two plots indicate that the model and simulation parameters used in the FDTD are sound. To calculate Figure 2b,c, a 20 nm × 20 nm × 2000 nm FDTD space with Bloch boundary conditions at the x and y directions and perfect matching layer (PML) boundary condition at the z direction was used. A mesh of z = 0.5 nm/pixel was used for the Au/Ti layers to sufficiently sample the thin Ti layer. The mesh size for the other areas in the x, y, and z directions was automatically optimized and set by the FDTD software. For Figure 2d–i, a 1500 nm × 1500 nm × 1500 nm FDTD space was used, while still maintaining both Bloch and PML boundary conditions at the x-y and z directions, respectively. Multiple meshes were used. The Ti layer mesh was set to z = 0.5 nm/pixel, while the Au layer and the space spanning a height of 150 nm from the surface of the Au layer mesh was set to z = 2 nm/pixel. For the other areas, the mesh size was set to x = y = 20 nm/pixel and z = 5 nm/pixel. The same values of refractive indices were used for both the analytical calculations for Figure 1 and the FDTD simulation.

Field enhancement of the evanescent field due to the excitation of surface plasmons induced by the presence of the Au/Ti layer is shown in Figure 2d–f in comparison to the evanescent field when there is no Au/Ti structure, as shown in Figure 2g–i. Field enhancement is especially high at the SPR angle, as shown in Figure 2e. Detuning by a few degrees shows significant decrease in the electric field. This is shown when comparing Figure 2c,d,f. Hence, measurement sensitivity is highest at the SPR angle. In principle, any change in the dielectric constant at a distance less than the decay length of the evanescent field (see Figure 2c) affects the propagation constant and is manifested as a shift in the reflectivity dip. What poses a challenge, though, is if there is a very small molecule adsorbed on the surface of the metal layer which induces a very small change in the refractive index. Very small refractive index changes could translate to negligible effect in the reflectivity measurement. To address this challenge, instead of measuring the intensity change, we propose to measure the position change of the reflected beam. Similar to SPR measurement, GH shift is also sensitive to the changes in the refractive index. Hence, any change in the refractive index on the sensing layer not only translates to intensity changes but also to position changes. In our scheme, we are able to amplify this position change by focusing the beam to a small beam waist.

**Figure 3:**
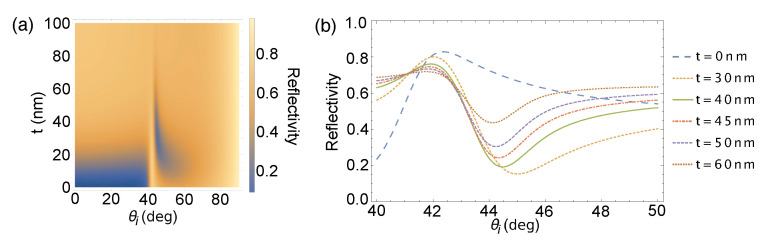
(**a**) Angular reflectivity spectra for a BK7/Ti/Au/air structure at varying Au film thicknesses with the incident beam tightly focused to a waist radius of 5.28 μm for an angular range from normal incidence to grazing incidence; and (**b**) the representative plots at several Au thicknesses.

Although the Fresnel-based equation could be used for loosely collimated beams, once the incident source is focused to a small beam waist and the divergence angle becomes larger, the Fresnel equations become convolved with the angular spectrum of the beam and hence are no longer sufficient. For such a kind of incident beam, a more general expression for the reflectivity based on the ratio of reflected power and incident power of a Gaussian beam is used given by [23]

(1) $$R_{G}\left(\theta_{i}\right)=\frac{w_{0}k_{1}}{\sqrt{2\pi }}\int_{-\infty }^{\infty }{\left|r\left(k_{x}\right)\right|}^{2}\exp\left[-\frac{w_{0}^{2}k_{1}^{2}}{2}{\left(\theta_{i}-\theta \right)}^{2}\right]\;d\theta ,$$

where $r(k_{x})$ is the angle-dependent Fresnel reflection coefficient [17], $w_{0}$ is the waist radius of the Gaussian beam propagating through a medium of refractive index $n_{1}$ and $k_{1}=2\pi n_{1}/\lambda$, and $\theta_{i}$ is the angle of incidence. Figure 3 shows the reflectivity spectra when the incident beam is focused to a small beam waist of 5.28 μm corresponding to our experimental configuration. Reflectivity values becomes averaged over the angular spread of the beam, leading to a decrease in the dip in the reflectivity spectrum and a shift in the location of the SPR angle. The shift in SPR angle is brought about by the change in propagation from the low efficiency of excitation of surface plasmons [24].

It is worthwhile to note that, although depolarization might occur due to the effective numerical aperture as determined by the beam waist, this effect is still negligible. Since GH shift is experimentally measured by obtaining the shift difference between *p* and *s* polarization, non-negligible depolarization could reduce the amount of measured shift. However, this is not the case for our experimental configuration since the depolarized field is much smaller than the remaining cross-polarized field from the polarization switching extinction by the electro-optic (EO) modulator (see Section 3.2).

The total GH shift Γ, measured by getting the location of the beam centroid at any propagation distance *z*, is the linear combination of the $\Delta_{GH}$ and $\Theta_{GH}$ shifts given by [9]

(2) $$\Gamma \left(z,\theta \right)=\Delta_{GH}\left(\theta \right)+z\Theta_{GH}\left(\theta \right).$$

Artmann’s formulation is a good approximation of $\Delta_{GH}$ shift. However, it breaks down for beams focused to a small beam waist since nonlinear phase terms can no longer be ignored for such cases [4,25,26]. A more accurate calculation of $\Delta_{GH}$ can be made by getting the location of the beam centroid at the focus plane where, for appreciably small beam waists, $\Delta_{GH}$ becomes negligible [4,15,25].

Bliokh and Aiello derived an expression for $\Theta_{GH}$ given by [1,9]

**Figure 4:**
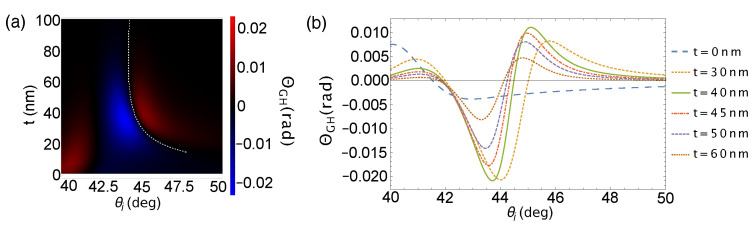
(**a**) $\Theta_{GH}$ spectrum for a BK7/Ti/Au/air structure with varying Au film thickness with tightly focused incident beam around the SPR angle; (**b**) and representative plots at several Au thicknesses.

(3) ΘGHα=−θ022Re∂lnrα∂θi,

where $\theta_{0}=\lambda /\pi w_{0}$ is the angular spread of the beam, and $r_{\alpha }=R_{\alpha }\exp\left(i\phi_{\alpha }\right)$ is the Fresnel reflection coefficient. The physically measured value of the angular change is $z\Theta_{GH}$. Using the Fresnel reflection coefficient, the partial derivative simplifies to a reflectivity-dependent term; as such, the beam waist dependence calculation of $\Theta_{GH}$ becomes straightforward with the use of Equation (1) for the reflectivity term. Figure 4 shows the Au film thickness dependence of $\Theta_{GH}$. $\Theta_{GH}$ is calculated to be zero at the SPR angle of each Au film thickness highlighted by the white dashed line in Figure 4a, but large magnitude of $\Theta_{GH}$ is shown within its vicinity (left and right of the white dashed line). The difference in the magnitudes of the positive and negative values is attributed to the asymmetry in the reflectivity plot.

Combining the resulting $\Delta_{GH}$ and $\Theta_{GH}$ shifts results in Γ being dominated by $\Theta_{GH}$ shift only. As such, calculations henceforth assume that $\Gamma \left(z,\theta \right)\approx z\Theta_{GH}\left(\theta \right)$.

## 3. Materials and Methods

### 3.1. Substrate and Monolayer Preparation

Substrates were prepared by electron beam (EB) evaporation of Au metal on UV/O${}_{3}$-cleaned 1-mm thick glass slides. Ti adhesion layer was deposited under a process pressure of 8.4 × 10${}^{-4}$ Pa at a rate of 0.3 Å/s until Ti thickness was 2.5 nm. Au films were then deposited on top of the Ti adhesion layer under 5.4 × 10${}^{-4}$ Pa at a rate of 1 Å/s until Au thickness was 47.5 nm. Both thicknesses were calibrated using a quartz crystal microbalance sensor. Although our calculations have indicated an optimum Au film thickness of 43.8 nm, we opted to use 47.5-nm thick Au film since fabrication of thinner films would have an increased surface roughness, which is detrimental for both BT-SAM formation and GH shift measurements.

We used two sample sets in this work, with each set consisting of four 10 mm × 10 mm substrates. The first sample set was a bare Au substrate. The second sample set was a Au substrate coated with a BT-SAM. Both substrates were subjected to cleaning with UV/O${}_{3}$ and sonication in acetone and ethanol to remove surface contaminants. BT-SAM was formed by immersing the Au substrate in 1 mM solution of BT (≥98%, Nacalai Tesque Inc., Kyoto, Japan) in ethanol for at least 18 h [27]. Samples were stored in ethanol until measurements were performed where they were rinsed with ethanol and dried in a stream of N${}_{2}$ just before measurement. Contaminants could adhere to the bare Au substrates even after removing from ethanol whereas BT-SAM coated Au substrates are expected to have less contamination. To keep the condition of the samples as identical as possible, particularly the level of contamination, and to eliminate any deviations caused by both environmental and experimental condition, two sample sets were used wherein measurements between sample sets were made within a short time interval. Both substrates came from the same batch of fabrication and cut from the same substrate which minimizes potential fabrication errors.

**Figure 5:**
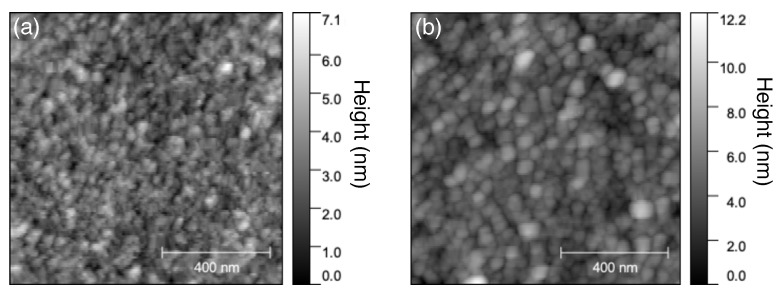
AFM image of (**a**) bare Au substrate and (**b**) Au substrate with BT. The scan area was set at 1 μm × 1 μm.

In our previous work, in Ref. [15], we used a relatively rough Au film surface prepared by radio frequency (RF) sputtering with substrate heating. Rough Au film would increase the surface area, hence, increase the number of adsorbed molecules, which is preferred for sensing applications. However, in this work, the Au film with smoother morphology is preferred to improve the formation of BT-SAM on the Au film surface. As such, Au film was prepared by EB evaporation without substrate heating giving a relatively smoother surface morphology, as shown in Figure 5a. The root mean square (RMS) roughness of the EB evaporation fabricated film is 0.84 nm—much smoother than the RF sputtered film with RMS roughness 5.67 nm [15]. The surface morphology is slightly changed with the formation of BT-SAM, as shown in Figure 5b, with an RMS roughness of 1.2 nm.

### 3.2. SPR and GH Shift Measurement

**Figure 6:**
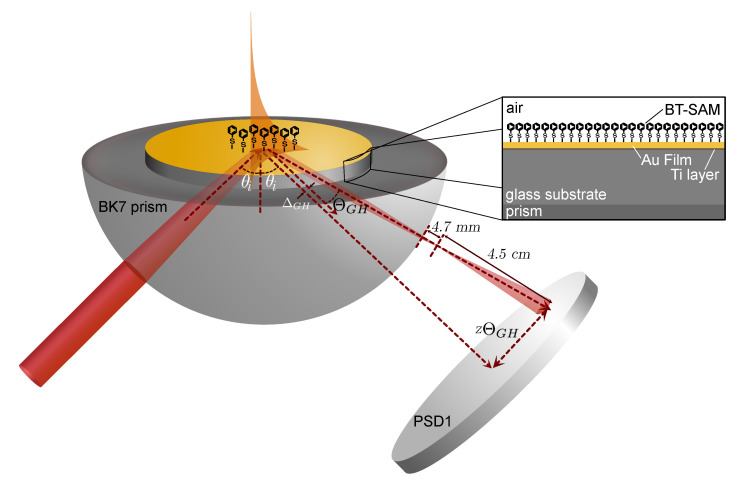
Schematic representation of the experimental setup and the sample geometry and its interaction with the incident beam.

The basic system configuration can be found elsewhere [15]. In brief, we used a linearly polarized helium-neon (HeNe, λ = 632.8 nm) laser. For *p*-*s* polarization switching, we used an EO modulator (Conoptics 350-80) with an extinction ratio better than 500:1 and at 1 kHz frequency modulation. A nonpolarizing beam splitter (NPBS) was used to separate the beam into a sample beam and a reference beam. The sample beam (75 μW) is directed toward a position sensing detector (Thorlabs PDP90A, PSD1) after reflection from the Au film mounted on a rotation stage (Sigma Koki SGSP-120YAW) with step size of 0.05${}^{\circ }$. PSD1 is positioned 4.5 cm from the beam waist, as shown in Figure 6, along the 2θ arm of the rotation stage. The reference beam is directed toward another PSD2 to monitor the voltage output of the incident beam.

SPR reflectivity measurements were obtained from the ratio of the voltage output of the two PSDs for the sample beam and the reference beam, whereas GH shift measurements were obtained by monitoring the displacement of the reflected beam using PSD1 and extracted using a lock-in amplifier (Stanford Research System SRS830) referenced by the EO modulator with 10 ms time constant. Since surface plasmon occurs under *p* polarized incidence, GH shift under *s* polarized illumination served as the reference.

Beam focusing was induced by the small diameter of the hemispherical BK7 prism (*D* = 20 mm, *n* = 1.515) to which the Au substrate was attached to using an index-matching oil (*n* = 1.518). Calculation of the beam propagation through the prism showed that, for an incident beam with a diameter of 560 μm characterized by a beam profiler (Gentec EO, Beamage-4M-Focus), the beam is focused 4.7 mm from the last interface of the surface of the prism to a waist radius of 5.28 μm and diverges at a large angle. This propagation of the beam is also shown schematically in Figure 6. As pointed out in Refs. [14,28,29], the focus spot does not need to be at the interface. Such is the case in our work where the beam is focused outside the hemispherical prism.

Proper care must be observed when setting the beam waist since smaller beam waist translates to larger angular spread. The finite size of the detector and its distance from the beam waist must be taken into account. Under the current setup, the beam diameter at PSD1 is much smaller than the active area of PSD1 (9 mm × 9 mm).

## 4. Results and Discussion

**Figure 7:**
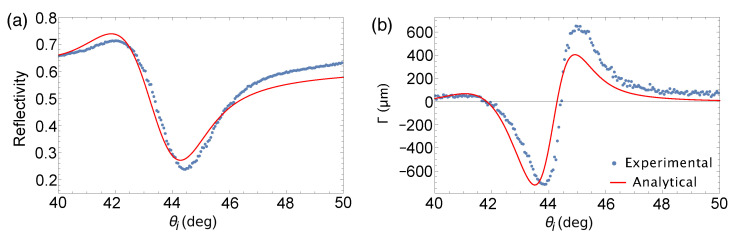
Comparison between analytical and experimental values of (**a**) reflectivity and (**b**) GH shift for a bare Au substrate under focused beam incidence.

Experimental results for reflectivity and GH shift are shown in Figure 7. The shallow dip of the experimental reflectivity plot, as shown in Figure 7a, is consistent with the analytical calculation under focused incidence indicating the decrease in Q factor from the larger spread of the wave vector. For a focused incident beam, the SPR angle calculated for a Au film ($t_{Au}$ = 47.5 nm) with Ti adhesion layer ($t_{Ti}$ = 2.5 nm) is at 44.28${}^{\circ }$. Our measurements showed an SPR angle of 44.45${}^{\circ }$. This deviation could be attributed to the hydrophilic nature of a clean Au substrate. Since measurements were performed in ambient, exposure of the Au substrate to atmospheric air could have introduced contamination on the Au surface. Specifically, Smith [16] pointed out that exposure of clean Au surface to the atmosphere showed oxygen and carbon contamination. The 0.17${}^{\circ }$ positive shift in the SPR angle with respect to the analytically calculated SPR angle which corresponds to a real bare Au surface is indicative of a change in the effective RI in the sensing layer. This RI change could have been induced by the contaminants on the Au surface. Similar observations were made in our previous work in Ref. [15]. In other words, the surface contamination determines the sensitivity limit of using a bare Au substrate in ambient without active control of its surface properties.

GH shift measurement in Figure 7b shows $\Theta_{GH}$-dominated feature corroborating with calculations shown in Figure 4b indicating that indeed $\Delta_{GH}$ becomes negligible for beams focused to a small beam waist. The SPR angle obtained from GH shift measurement for which Γ=0 is at $\theta_{i}=$44.43°, which is almost equal to the SPR angle determined from the reflectivity measurement and in good agreement with Figure 4. We measured a positive extremum value of 652.4 μm at 44.95° and a negative extremum value of −715.3 μm at 43.85°. The positive GH shift indicates movement of the beam to an angle lesser than the SPR angle, and the negative GH shift implies movement in the opposite direction.

The enhancement of GH shift is induced by the rapid change in both reflectivity and phase brought by the excitation of surface plasmons. As such, the amount of enhancement is determined by how well surface plasmons are excited as indicated by the Q factor. Essentially, materials with high Q factor would induce larger GH shift. In addition, the amount of beam focusing also affects the amount of GH shift enhancement. Typical measurements make use of nearly collimated beams which would induce large $\Delta_{GH}$-dominated GH shift. However, as the incident beam is focused, the reflected beam becomes distorted resulting to a deflection. The tighter the incident beam is focused, the more the reflected beam becomes deflected resulting to a $\Theta_{GH}$-dominated GH shift. But as the angular spread of the beam increases, the efficiency of surface plasmon excitation decreases. These consequences present a trade-off between the Q factor and the enhanced $\Theta_{GH}$ measurement. Nonetheless, we were able to show in Ref. [15] that, even if the Q factor is low, the measured GH shift is still significantly large. Likewise, in this work, we show significantly large GH shift within the vicinity of the SPR angle, reaching a magnitude of around 700 μm.

**Figure 8:**
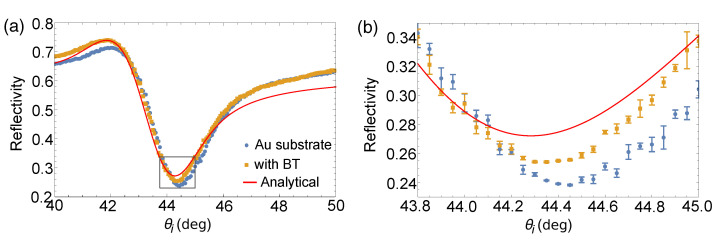
(**a**) Comparison of experimentally obtained reflectivity plots of bare Au substrate (blue circle); Au substrate coated with BT-SAM (yellow square) with analytical calculation (red solid line). (**b**) Closer inspection of boxed area in (**a**) over a smaller angular range to show the shift in $\theta_{SPR}$.

With the presence of BT-SAM on the Au substrate to make the surface hydrophobic, we were able to show a shift in the SPR angle manifested in both reflectivity measurement and GH shift measurement. Figure 8 shows the reflectivity plots for both bare Au substrate and Au substrate with BT-SAM compared with the plot based on analytical calculation for a BK7/Ti/Au/air structure. The SPR angle measured for the BT-SAM covered Au substrate was at 44.35°. In comparison to the analytical value, the larger SPR angle shift measured for the bare Au substrate could be attributed to the surface contaminants, while the smaller SPR angle shift measured for the BT-SAM covered Au substrate could be due to the presence of the molecular monolayer.

**Figure 9:**
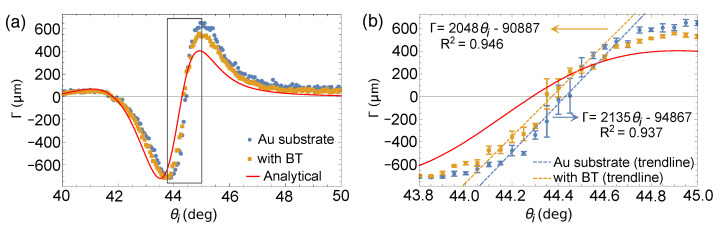
(**a**) GH shift of bare Au substrate and Au substrate coated with BT-SAM in comparison with analytical calculation. (**b**) GH shift within the vicinity of $\theta_{SPR}$ (area enclosed by box in (**a**)). The linear trendlines in (**b**) were used to extract $\theta_{SPR}$ from GH shift measurement.

Figure 9 shows the GH shift measurements of the bare Au substrate and with the presence of BT-SAM. Without any loss of generality, we took the linear regression within the vicinity of Γ = 0, before Γ reaches its maximum and minimum value, to determine a precise value of $\theta_{SPR}$. Trendlines are shown in Figure 9b, as well as their corresponding curve fitting equations. $R^{2}$ values indicate good linear correlation among chosen data points. We believe that linear regression within the vicinity of the SPR angle is a simplistic approach in determining the SPR angle. Our values are consistent with $\theta_{SPR}$ obtained from reflectivity measurement. From the linear regression, we determined $\theta_{SPR}$ = 44.43° for the bare Au substrate and $\theta_{SPR}$ = 44.37° when BT-SAM is present. Error bars shown in Figure 8b and Figure 9b correspond to the standard error of the mean obtained from measurements from four different substrates used in each sample set. Small error bars indicate repeatable measurements for samples from the same fabrication batch.

It may seem counter-intuitive that the SPR angle shifted to a smaller angle when comparing the experimental measurements for bare Au substrate and with BT-SAM shown in both reflectivity and GH shift measurements in Figure 8 and Figure 9, respectively. However, this is consistent with the discussion for the bare Au substrate highlighting its hydrophilic nature. The presence of BT-SAM renders the Au substrate hydrophobic. As such, reflectivity and GH shift measurements, even in ambient, show plots much closer to the analytical plot for bare Au substrate since the presence of BT-SAM made the sample less prone to atmospheric contamination. The <0.1° positive shift of the experimentally measured SPR angle with respect to the calculated SPR angle could have been induced by the presence of BT-SAM itself.

The main result in this work is the confirmation of the presence of BT-SAM on the Au substrate based on the surface modification induced as evidenced by the changes in both reflectivity and GH shift measurements. Since measurements were performed in ambient, we cannot eliminate the presence of contamination on the Au substrate incurred during measurement. Nonetheless, we have demonstrated that the presence of BT-SAM unambiguously altered the surface property which affected both reflectivity and GH shift measurements. We suppose that a more controlled environment during measurement, e.g., measurement performed in liquid environment, could be implemented that would potentially eliminate contamination during measurements. This is the next phase of our work and is currently in development.

## 5. Conclusions

The hydrophilic nature of a bare Au substrate makes it susceptible to contaminants. Using SPR and $\Theta_{GH}$ shift measurements, we showed that these contaminants increased the measured SPR angle from its expected value. The large deviation in the SPR angle was minimized with the formation of BT-SAM on the Au substrate since it made the surface hydrophobic. For the BT-SAM covered Au substrate, the small change in the measured SPR angle from the expected value measured in both the SPR and $\Theta_{GH}$ shift measurement could indicate the detection of the molecular monolayer itself. Quantification of the amount of BT-SAM formed on the Au surface requires measurements in a controlled environment, which is the next phase of this work. Furthermore, inhibition of surface contamination would improve the sensitivity limit of using SPR-enhanced GH shift measurement as a sensor.

## Data Availability

The data presented in this study are available on request from the corresponding author.

## Abbreviations

The following abbreviations are used in this manuscript:

GH	Goos-Hänchen
SPR	surface plasmon resonance
RI	refractive index
SAM	self-assembled monolayer
BT	benzenethiol
BT-SAM	benzenethiol self-assembled monolayer
TMM	transfer matrix method
FDTD	finite-difference time-domain
PML	perfect matching layer
EO	electro-optic
EB	electron beam
RF	radio frequency
RMS	root mean square
NPBS	non-polarizing beam splitter
PSD	position sensing detector
NDF	neutral density filter
